# Comparison of growth and nutritional status in infants receiving goat milk–based formula and cow milk–based formula: a randomized, double-blind study

**DOI:** 10.3402/fnr.v59.28613

**Published:** 2015-12-10

**Authors:** Meihong Xu, Yibin Wang, Zhiyong Dai, Yanchun Zhang, Yong Li, Junbo Wang

**Affiliations:** 1 Department of Nutrition and Food Hygiene, School of Public Health, Peking University, Beijing, China; 2 Beijing Key Laboratory of Toxicological Research and Risk Assessment for Food Safety, Peking University, Beijing, China; 3 Ausnutria Hyproca Dairy Group BV, Changsha, China

**Keywords:** infant, formula, goat milk, growth, nutritional status

## Abstract

**Objective:**

To compare the growth and nutritional status of infants fed goat milk–based formula (GMF) and cow milk–based formula (CMF).

**Methods:**

The study was conducted in Beijing, China. It was a double-blind randomized controlled trial. A total of 79 infants aged 0–3 months old were recruited and randomized in GMF or CMF group. The infants were fed the allocated formula to 6 months. The weight, length, and head circumference were measured at the enrolment, 3 and 6 months. The start time and types of solid food were recorded. Blood elements, urinal, and fecal parameters were also tested.

**Results:**

The average weight of infants in the GMF group (mean±SD) was 4.67±0.99 kg and in the CMF group 4.73±1.10 kg at enrolment, and 8.75±0.98 kg (GMF) and 8.92±0.88 kg (CMF) at 6 months. There were no differences in the adjusted intention-to-treat analyses of weight, length, head circumference, and BMI *z*-scores between the two formula-fed groups over the 6-month study. Similarly, there were no remarkable differences in the timing and types of solid food, blood elements, urinal, and feces parameters, between the GMF and CMF group. No group differences have been shown in bowel motion consistency, duration of crying, ease of settling, or frequency of adverse events.

**Conclusions:**

GMF-provided growth and nutritional outcomes did not differ from those provided by CMF.

Goat milk is a food of high nutritional value, with high biological value protein, and a good source of short and medium chain fatty acids, minerals, and vitamins (1). The fat from this product has better digestibility, the protein has smaller allergenic potential, and this milk also has less lactose than cow milk (1). Furthermore, goat milk provides a better use of iron, which minimizes possible interactions between iron and other minerals such as calcium, phosphorus, and magnesium (2–6). More than a simple source of essential nutrients, goat milk contains many functional components, including lactoferrin, oligosaccharides, nucleotides, taurine, polyamines, and bioactive peptides (1, 7). Developing new products that mimic human milk properties is a current target for infant formula manufacturers. For the above, beneficial, goat milk–based formula (GMF) emerged as a new candidate.

When discussing GMF according to the existing literature, we found that they were based mainly on the following points: firstly, the topics are usually on the comparison of nutrition content among the goat/sheep, cow, and human milk (8–11). Secondly, the biological effect of GMF has not been clear yet. Thirdly, the subject of animal studies is limited in the anti-inflammation, anti-allergy, anemia-healing, and the absorption of minerals, amino acids, etc. (12–16). Few studies are clinical trials (17, 18). These studies conducted in Australia showed that the growth of infants fed a goat milk infant formula was similar to infants fed a whey-based cow milk infant formula. However, the nutritional outcomes were limited.

Therefore, the aims of this study were to determine whether growth differed significantly for infants fed GMF in China, compared with cow milk–based formula (CMF).

## Subjects and methods

### Study design and participants

This was a single center, prospective, double-blind, randomized, and controlled comparison of commercially available infant formulas: GMF and CMF. Ethical approval was obtained from the Biomedicine Ethics Committee of Peking University (IRB00001052-11036). Written informed consent was obtained from the mothers of all enrolled infants.

Infants were eligible for inclusion in the study if the following criteria were met: 1) mother was primipara; 2) a healthy term infant with gestation of 37–42 weeks and birth weight ≥2.5 and ≤4.75 kg; and 3) aged up to 3 months. Infants were excluded if 1) they were from multiple births or had severe congenital or metabolic disease likely to affect feeding or growth; 2) mothers who had a spontaneous abortion, pregnant in feeding period, or taking drugs and/or food allergies; and 3) the infant or mother had participated in other clinical trials in the past 4 weeks. Infants were identified and referred by doctors in the postnatal wards at Fengbo Health-center in Shunyi District, Beijing, China.

### Sample size, randomization, and masking

As no local reference data on growth variance were available, sample size requirements were estimated based upon published contemporary growth studies of infants fed milk formula. A sample size of 60, 30 in each group, was expected to provide 80% powder (with *α*=0.05) to detect a 4 g/day difference between the GMF and CMF groups in bodyweight.

Eligible formula-fed infants were randomly assigned to receive either GMF or CMF group. From the randomization list, a unique identifying code was created for each enrolled infant and used to label all the formulas for that infant. The researcher sent these codes to the formula-packaging company, instructing the packing company on which unique codes were to be applied to boxes of GMF and CMF. Each enrolled infant, therefore, had an individually coded supply of infant formula. The investigators, who were the only people who allocated boxes of formula powder to each infant, were kept blinded with respect to which infant codes were for either GMF or CMF. The code linking each infant's identifying number with milk formula type was not broken until after the last infant had completed the study.

### Experimental product

The GMF and CMF were both manufactured and provided by Ausnutria Hyproca Dairy Group BV. The nutritional composition of both formulas (GMF and CMF) is given in Table 1.

**Table 1:** Nutritional composition of the two infant formulas used in the study

Nutritional composition	GMF (100 g)	CMF (100 g)
Energy (kJ)	2,012	2,131
Protein (g)	10.5	11
Fat (g)	25.3	27
1, 3-Dioleoyl-2-palmitoyl glyceride (g)	4.7	–
Linoleic acid (g)	4.7	4.2
α-linolenic acid (mg)	499	420
Arachidonic acid (mg)	100.4	60
DHA (mg)	50.8	40
Carbohydrate (g)	57.1	55.2
Lactose (g)	52	≥49.68
Fructo-oligosaccharide (mg)	1,200	350
Galacto-oligosaccharides (mg)	700	410
Minerals		
Ca (mg)	438	362
P (mg)	240	245
Mg (mg)	41	30
Fe (mg)	3.8	4.65
Zn (mg)	3.2	3.75
Cu (µg)	331	330
K (mg)	550	380
Cl (mg)	482	320
Vitamins		
Vitamin A (IU)	1,508	1,498
Vitamin C (mg)	69	56
Vitamin B1 (µg)	667	500
Vitamin B2 (µg)	1,309	650
Vitamin B6 (µg)	468	350
Vitamin B12 (µg)	1.9	1.5
Niacin (µg)	3,810	4,000
Folic acid (µg)	67	54.1
Pantothenic acid (µg)	2,993	2,800
Biotin (µg)	31	13.8
L-carnitine (mg)	15.6	–
Taurine (mg)	41.5	35
Nucleotide (mg)	22.1	

### Procedures

The parents of formula-fed infants were asked to feed their infants the allocated study formula from enrolment to 6 months. Study formulas were supplied free of charge until the end of the study.

The primary outcomes were infant weight, length, and head circumference, measured at enrolment, 3 months, and 6 months. All anthropometric growth data were converted to *z*-scores using WHO Child Growth Standards (www.who.int/childgrowth/en/). Secondary outcomes included nutritional status and general health.

At each growth assessment time point, parents were asked through a structured interview whether their infants had experienced any health problems including respiratory illness, gastrointestinal illness, reflux, eye infection, ear, nose and throat conditions, fever, urinary tract infection, and thrush. Serious adverse events, defined as death or hospital admission for more than 24 h during the 6-month study period, were also recorded.

An adverse event included any illness, sign, symptom, or clinically significant laboratory test abnormality that appeared during the course of the trial, irrespective of any potential relationship this event may have had with the trial formulas. Infants experiencing adverse events that caused discontinuation of the study formula received follow-up. With the mother's permission, the subsequent scheduled visits were completed and measurements of weight, length, and head circumference were taken.

A serious adverse event was defined as any untoward medical occurrence that resulted in death, life-threatening illness, hospitalization, serious disability, congenital anomaly, or required intervention to prevent permanent impairment or damage. Serious adverse events were reported immediately to the project manager, the principal investigator, and the sponsor and were notified to the researchers and the Ethics Committee.

The researchers received unblinded data from the trial at monthly intervals. They reviewed the non-serious and serious adverse events and made recommendations on study continuation to the principal investigator.

### Outcome assessments

The study nurse visited the infant at the enrolment, 3 months, and 6 months of age. At each visit, infant formula was provided, adherence with the study requirements determined, the infant measured, the diary reviewed, and the number of unused sachets recorded. Adverse events, other foods and drinks consumed, the infant's typical bowel motions, usual sleeping and crying patterns, and prescribed medicines were recorded.

The timing of introduction of solids about 4 and 6 months was at the discretion of the families for infants. The start time and types of solid food were recorded.

A small non-fasting peripheral blood sample (20 µL) was collected to assess blood elements at enrolment, 3 and 6 months by flame atomic absorption spectrometry.

Parents were asked to assess stool frequency consistency. Urine and feces were also analyzed at enrolment, 3 and 6 months. The protein content of feces was determined using Coomassie brilliant blue. EILSA method for testing the sIgA in feces at enrolment, 3 and 6 months.

### Other assessments

Demographic and baseline characteristics, including infant sex, weight, and length at birth, age at enrolment, and anthropometric measurements at enrolment and maternal age, parity, and history of smoking and drug, and alcohol use during pregnancy, were recorded at trial entry.

### Statistical analyses

Descriptive statistics include mean (standard deviation, SD) or median (interquartile range) for continuous variables and counts (percentage) for categorical variables. Equality and normality of variance were checked prior further analysis, and variables with a skewed distribution were log transformed.

Statistical analyses were performed using SPSS software (version 19.0; SPSS, Inc., Chicago, IL). Variances in the measurement data were checked for homogeneity by Bartlett's test. When the data were homogeneous, the independent samples *t* tests and chi-squared test were used. All reported *P* values were two-sided. A value of *P<*0.05 was considered significant.

## Results

**Fig. 1:**
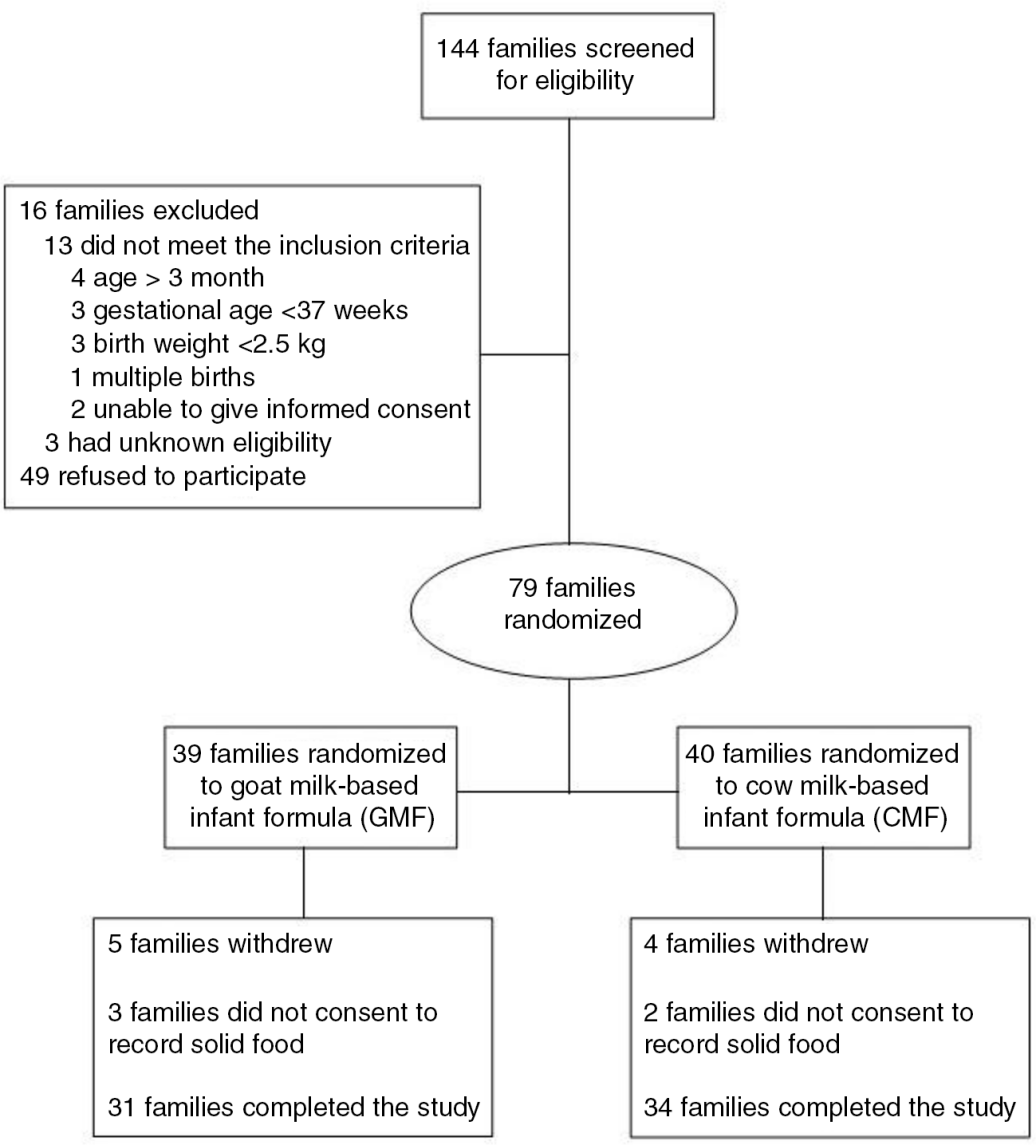
Flow diagram of participants’ progress through the trial.

The participants were recruited between October 2011 and July 2012 at Fengbo Health Center. Of the 144 families who were approached to participate in the study, 65 were eligible and 79 (39%) consented. A total of 39 infants were GMF and 40 were CMF. More details are given in the flow chart of study participant selection in Fig. 1.

### Baseline characteristics

Maternal characteristics as well as infant anthropometrics at birth and at study entry are summarized in Table 2. The mean age of infants at study entry was 39.23±19.59 days and 50.8% were male. The baseline characteristics of the participants were comparable between GMF and CMF. The blindness index, which indicates the percentage of mothers who guessed their treatment group correctly above chance, was 10.3% for the GMF group compared with 12.5% for the CMF group.

**Table 2:** Characteristics of the participants

	GMF	CMF	*P*
Maternal characteristics			
Age (years)	29.46±4.15	29.69±4.54	0.83
Education			0.71
Secondary incomplete	6 (15.4)	9 (22.5)	
Certificate/diploma or secondary complete	13 (33.3)	13 (32.5)	
Degree or higher degree	20 (51.3)	18 (45.0)	
Smoking during pregnancy	1 (2.6)	2 (5.0)	0.54
Drinking during pregnancy	0 (0.0)	0 (0.0)	0.35
Infant birth characteristics			
Sex, male	16 (41.0)	17 (42.5)	0.54
Birth weight (kg)	3.26±0.46	3.28±0.58	0.89
Birth length (cm)	47.65±8.26	49.73±1.62	0.15
Infant baseline data			
Age at enrolment (days)	42.65±22.09	35.12±17.51	0.131
Weight at enrolment (g)	4.67±0.99	4.73±1.10	0.83
Length at enrolment (cm)	55.65±3.63	54.35±4.06	0.18
Head circumference at enrolment (cm)	37.26±1.94	37.17±2.99	0.86

Data were mean value (standard deviation) or *n* (percentage). *P* values are for the independent *t* test or non-parametric test or chi-squared test for means of two groups (*a=*0.05). GMF, goat milk–based infant formula; CMF, cow milk–based infant formula.

Compliance with the definition of GMF or CMF from enrolment to 6 months after intervention was observed in 31 (79.5%) of the 39 infants in GMF group and 34 (85%) of the 40 infants in CMF group. The level of compliance in GMF group was not significantly different from that in CMF group (*P>*0.05).

### Growth

There were no differences in the adjusted intention-to-treat analyses of weight, length, head circumference, and BMI *z*-scores between the two formula-fed groups over the 6-month study (Table 3). Also, gains in weight, length, or head circumference from registration did not differ between GMF and CMF groups.

**Table 3:** Weight, length, head circumference, and BMI *z*-scores of infants fed GMF and CMF

	GMF	CMF	*P*
3 months after intervention			
Weight *z*-score	0.72±1.25	0.24±1.03	0.10
Length *z*-score	0.35±1.51	0.38±1.46	0.95
Head circumference *z*-score	0.11±1.23	−0.13±1.11	0.32
BMI *z*-score	0.68±1.16	0.74±0.96	0.83
6 months after intervention			
Weight *z*-score	0.46±1.06	0.23±1.20	0.43
Length *z*-score	0.34±1.53	0.55±1.26	0.56
Head circumference *z*-score	0.06±1.00	−0.32±1.34	0.22
BMI *z*-score	0.42±1.08	0.68±0.93	0.44

*z*-Score data were based on WHO reference data; data were mean value (standard deviation). *P* values are for the independent *t* test or non-parametric test for means of two groups (*a*=0.05). GMF, goat milk–based infant formula; CMF, cow milk–based infant formula.

The daily intake of study formula ranged from 76.88±43.98 g at the enrolment to 173.40±71.35 g at 6 months after intervention. Similarly, there were no remarkable differences in formula consumption and the days of anti-feedant (data not shown).

### The timing and types of solid food

There were no differences in the timing of solid food between GMF and CMF group, which was 130.50±34.29 and 124.56±33.25 days, respectively. Meanwhile, the timing of various types of solid food did not show differences between the two groups. The timing of various types of solid food, such as cereal, eggs, vegetables, fruit, and meat, were 140.25±34.50, 136.60±37.79, 157.25±38.45, 136.62±32.51, and 206.00 days in GMF groups, respectively; whereas 132.50±29.81, 133.88±32.30, 198.20±19.27, 152.89±28.87, and 172.00 days in CMF groups, respectively.

### Blood elements status

**Fig. 2:**
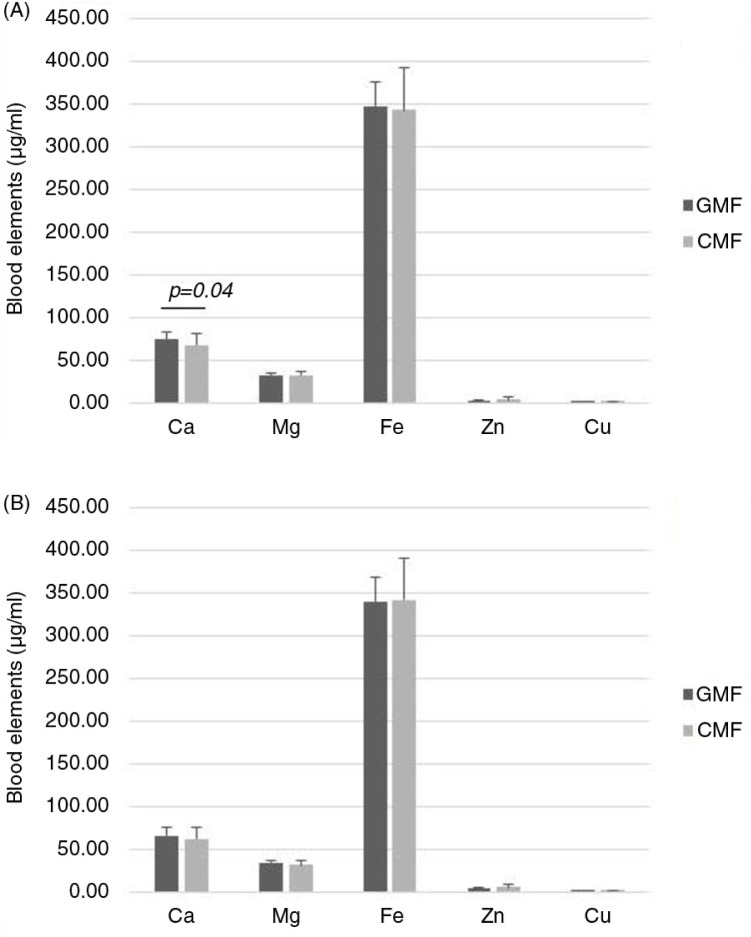
Concentrations of blood elements in the plasma of infants fed GMF or CMF: (A) male and (B) female. GMF, goat milk–based formula; CMF, cow milk–based formula; values are means, with standard deviations represented by vertical bars.

As shown in Fig. 2, there were no differences in blood elements between the two formula-fed groups (*P>*0.05), with the exception of Ca at the end of 3 months after intervention (*P=*0.04).

### Urine and feces indexes

As shown in Table 4, there were no significant differences in urinal and fecal parameters between the two formula-fed groups (*P>*0.05).

**Table 4:** Urinal and fecal parameters of infants fed GMF or CMF

	GMF	CMF	*P*
At the enrolment			
UBG (mmol/L)	3.40±0.00	3.40±0.00	1.00
SG	1.01±0.01	1.01±0.01	0.61
U-PH	6.24±0.84	6.09±0.61	0.43
U-BLD (cell/mL)	0.00±0.00	0.00±0.00	
U-PRO (g/L)	0.00±0.00	0.00±0.00	
U-WBC (cell/mL)	0.00±0.00	0.00±0.00	
U-GLU (mmol/L)	0.00±0.28	0.00±0.00	
U-Vit (mmol/L)	0.08±0.21	0.04±0.15	0.33
F-WBC (cell/mL)	0.00±0.00	0.00±0.00	
F-RBC (cell/mL)	0.00±0.00	0.00±0.00	
F-PRO (mg prot/g)	13.11±7.03	15.67±14.57	0.40
F-sIgA (mg/g)	0.04±0.02	0.04±0.02	0.78
3 months after intervention			
UBG (mmol/L)	3.40±0.00	3.40±0.00	1.00
SG	1.01±0.00	1.02±0.01	0.78
U-PH	6.60±0.51	6.22±0.47	0.00
U-BLD (cell/mL)	0.00±0.00	0.00±0.00	
U-PRO (g/L)	0.00±0.00	0.00±0.00	
U-WBC (cell/mL)	0.00±0.00	0.00±0.00	
U-GLU (mmol/L)	0.00±0.00	0.00±0.00	
U-Vit (mmol/L)	0.18±0.55	0.14±0.37	0.77
F-WBC (cell/mL)	0.00±0.00	0.00±0.00	
F-RBC (cell/mL)	0.00±0.00	0.00±0.00	
F-PRO (mg prot/g)	10.22±5.29	13.47±7.28	0.06
F-sIgA (mg/g)	0.05±0.01	0.04±0.01	0.13
6 months after intervention			
UBG (mmol/L)	3.40±0.00	3.40±0.00	1.00
SG	1.02±0.00	1.02±0.00	0.12
U-PH	6.24±0.46	6.14±0.53	0.43
U-BLD (cell/mL)	0.34±1.84	3.59±14.71	0.24
U-PRO (g/L)	0.00±0.00	0.01±0.03	
U-WBC (cell/mL)	2.41±13.00	15.63±88.39	0.35
U-GLU (mmol/L)	0.00±0.00	0.00±0.00	
U-Vit (mmol/L)	0.08±0.21	0.14±0.52	0.56
F-WBC (cell/mL)	0.00±0.00	0.09±0.53	
F-RBC (cell/mL)	0.00±0.00	0.00±0.00	
F-PRO (mg prot/g)	8.95±5.96	11.92±6.80	0.09
F-sIgA (mg/g)	0.05±0.01	0.05±0.01	0.19

Data were mean value (standard deviation). *P* values are for the independent *t* test or non-parametric test for means of two groups (*a=*0.05). GMF, goat milk–based infant formula; CMF, cow milk–based infant formula; UBG, urobilinogen; SG, urine specific gravity; U-PH, urine pH; U-BLD, urine occult blood; U-PRO, urine protein; U-WBC, urine albumin; U-GLU, urine glucose; U-Vit, urine vitamin; F-WBC, feces albumin; F-RBC, feces red blood cell; F-PRO, feces protein; F-sIgA, feces sIgA.

### General health-related outcomes

There were no differences in the risk of an adverse health condition, including respiratory illness, gastrointestinal illness, reflux, eye infection, ear, nose and throat conditions, fever, urinary tract infection, and thrush between the GMF and CMF group, during the 6-month study period. The proportion of infants who had any serious adverse events during the study period was similar between GMF 6/39 and CMF 7/40 groups (two diarrhea and four throat conditions in GMF; one eczema, three diarrhea, two throat conditions, and one running nose in CMF). The most common serious adverse events were bronchiolitis and other respiratory infections. No infants died.

## Discussion

The aim of the present study is to evaluate, in healthy term infants, the effect of feeding GMF for 6 months on growth, nutritional status, tolerance to formula, and a wide range of health-related outcomes in a randomized controlled trial, comparing with CMF. We detected no difference in *z*-scores for infant weight, length, head circumference, and BMI between the two formula-fed groups in the 6-month study. This is consistent with the previous study (17, 18).

Blood, urine, and feces samples were collected from both groups. There were minor differences in the blood Ca between the formula-fed groups at 3 months. This is consistent with the previous study (12, 13, 19). It may be related to the influence of GMF on the digestive and metabolic utilization of calcium and it is probably reflected in differences in composition of the two formulas. For instance, CMF contained Ca 438 mg/100 g, whereas GMF contained Ca 362 mg/100 g. Concentrations of blood elements measured at 3 and 6 months were within the normal reference range for infants of this age. No significant differences have been found between the two infant groups in urine and feces indicators. It was consistent with the Australia study (17). In their study, the frequency of bowel motions in the GMF group was not excessive and was not associated with any significant difference in consistency. However, beta-diversity analysis of total microbiota sequences and Lachnospiraceae sequences revealed that they were more similar in breast milk/goat milk comparisons than in breast milk/cow milk comparisons (20).

No differences were noted between the two groups in infant behaviors or frequency of adverse events, such as vomiting, diarrhea, constipation, food refusal, or screaming. Therefore, the tolerability and safety of GMF did not appear to be any different from CMF.

It must be noted that this study was not designed to determine differences in serum allergenicity between GMF and CMF. Children with proven immunoglobulin E (IgE)-mediated CMF allergy are also at increased risk of allergy to GMF (21). In such children, GMF would be inappropriate. We determined the level of feces sIgA, which did not show any differences between the two infant groups. Therefore, in healthy non-allergic children, the data from this study indicate that GMF is a suitable alternative to CMF. Moreover, a breast milk-fed group was not included in this trial.

## Conclusions

In particular, this study shows that adequate growth and nutritional status is sustained when GMF is the predominant source of nutrition. It also showed that there were no differences between the GMF and CMF group. Breast milk remains the food of choice for infants, but for infants who cannot be breast-fed and who are allergic to cow milk, this study shows that GMF is an appropriate alternative. Further research is needed to use specific models to elucidate the effect of GMF on neurobehaviors or allergies.
